# Structural insights into the human PA28–20S proteasome enabled by efficient tagging and purification of endogenous proteins

**DOI:** 10.1073/pnas.2207200119

**Published:** 2022-07-19

**Authors:** Jianhua Zhao, Suraj Makhija, Chenyu Zhou, Hanxiao Zhang, YongQiang Wang, Monita Muralidharan, Bo Huang, Yifan Cheng

**Affiliations:** ^a^Department of Biochemistry and Biophysics, University of California, San Francisco, CA 94143;; ^b^UC Berkeley–UCSF Joint Graduate Program in Bioengineering, University of California, San Francisco, CA 94143;; ^c^Howard Hughes Medical Institute, University of California, San Francisco, CA, 94143;; ^d^Department of Cellular Molecular Pharmacology, University of California, San Francisco, CA 94143;; ^e^Department of Pharmaceutical Chemistry, University of California, San Francisco, CA 94143;; ^f^Chan Zuckerberg Biohub, San Francisco, CA 94158

**Keywords:** single-particle cryo-EM, proteasome, endogenous protein tagging, CRISPR-Cas9

## Abstract

The ability to produce folded and functional proteins is a necessity for structural biology and many other biological sciences. This task is particularly challenging for numerous biomedically important targets in human cells, including membrane proteins and large macromolecular assemblies, hampering mechanistic studies and drug development efforts. Here we describe a method combining CRISPR-Cas gene editing and fluorescence-activated cell sorting to rapidly tag and purify endogenous proteins in HEK cells for structural characterization. We applied this approach to study the human proteasome from HEK cells and rapidly determined cryogenic electron microscopy structures of major proteasomal complexes, including a high-resolution structure of intact human PA28αβ–20S. Our structures reveal that PA28 with a subunit stoichiometry of 3α/4β engages tightly with the 20S proteasome. Addition of a hydrophilic peptide shows that polypeptides entering through PA28 are held in the antechamber of 20S prior to degradation in the proteolytic chamber. This study provides critical insights into an important proteasome complex and demonstrates key methodologies for the tagging of proteins from endogenous sources.

## Significance

We report an approach of combining CRISPR-Cas9 gene editing with florescence cell sorting to tag and purify human endogenous protein complexes from HEK cells for structural studies by single-particle cryogenic electron microscopy. This procedure is demonstrated by applying the method to study human proteasomal complexes, enabling rapid determination of high-resolution structures of several proteasomal complexes. We envision this approach will enable structural, biochemical, and biophysical studies of many important endogenous human macromolecular complexes.

Recent technological breakthroughs in single-particle cryogenic electron microscopy (cryo-EM) have greatly accelerated the pace of high-resolution structure determination of biological macromolecules. However, a major bottleneck in the structural study of important targets including membrane proteins, protein–DNA complexes, and large protein assemblies is sample production. The challenge of producing functional proteins extends beyond structural biology and impacts many areas of biological sciences that currently rely on techniques of protein overexpression. Conventional methods of protein production involve overexpression of a target gene from a plasmid within heterologous systems, including bacterial (*Escherichia coli*), insect (Sf9), and mammalian (HEK) cells. Overexpression approaches frequently encounter issues with proteins that are misfolded, nonfunctional, or degraded by the host cells. Overexpression of certain proteins may also alter protein homeostasis within cells. Substantial optimization is often necessary to identify the right conditions to produce suitable proteins for study, which is time-consuming and challenging in many cases. The problem is compounded further by multiprotein complexes where multiple subunits need to be assembled in the correct order and stoichiometries, making the coexpression of multiple subunits and purification of protein assemblies even more challenging.

Studies of challenging proteins and large protein complexes have sometimes been possible by extracting samples from natural sources. While this approach has proven successful in special cases, examples of native proteins purified in this way have been largely limited to highly abundant and large protein assemblies that can be isolated by sucrose gradient ultracentrifugation (1) or where a known binding partner can be used as bait (2, 3). A general and efficient method to isolate proteins from endogenous sources with high specificity and at sufficient quantities for structural studies remains a goal of substantial interest. A rapid and efficient approach to add an affinity tag onto a target protein in human cells for affinity purification of endogenous complexes would greatly accelerate structural characterization of challenging and biomedically important targets. Furthermore, this strategy could be combined with complementary approaches such as mass spectrometry to better understand protein function under more native-like conditions.

Recent technological advances in CRISPR-Cas–mediated genome engineering have provided new avenues to target endogenous proteins from native sources by genetically incorporating an affinity tag onto a protein of interest for purification and downstream analysis (4, 5). However, the time-consuming process of generating and selecting gene-edited cells with an endogenously tagged protein has created a substantial barrier for this approach to be widely adopted by the structural biology community and other fields of protein science. A simple, fast, and minimally perturbing way to target and extract endogenous proteins would make this approach more widely accessible to the general scientific community. Here we describe a simple and highly efficient method that leverages the unique properties of split fluorescent proteins (6) to select cells harboring an affinity tag on target endogenous proteins for purification.

We demonstrate this approach on the study of the human proteasome. The proteasome plays an essential role in protein degradation and is critical to maintaining cellular protein homeostasis (7). Changes to proteostasis in cells and lowered protein degradation due to decreases in proteasomal activity have been linked to aging and neurodegeneration (8). While the proteasome has been a topic of extensive studies using a variety of strategies (9–12), the study of some human proteasomal complexes has remained challenging. In this study, by tagging, purifying, and determining structures of various human proteasomal complexes from HEK cells we demonstrate that the efficient protein tagging method presented here can facilitate rapid structural studies of endogenous human protein complexes in HEK cells. Analyzing cryo-EM structures of PA28–20S complexes reveals insights into PA28 stoichiometry and sheds light on how unfolded polypeptide substrates traverse through the antechamber of the 20S core particle before entering the proteolytic chamber for degradation.

## Results

### Rapid Tagging and Purification of Endogenous Proteins in HEK Cells.

**Fig. 1.**
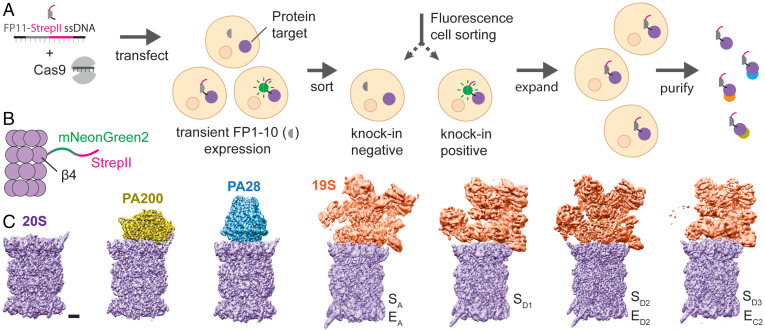
Tagging and purification of endogenous proteins for structural studies. (*A*) Single-stranded DNA encoding FP11-StrepII is delivered into the nucleus of HEK cells along with Cas9 protein complexed with gene-specific gRNA. Subsequent DNA cleavage and repair results in a mixed population of cells with and without FP11-StrepII successfully incorporated at the target site. Transient expression of FP1-10 in the cells enables conjugation between FP1-10 and FP11 to form fluorescent molecules, allowing rapid isolation of cells containing the tag by FACS. The isolated cell population can be expanded without the need for single-cell clonal selection to allow rapid scaling up of cultures for structural studies. Additionally, the cells are expanded in the absence of FP1-10, resulting in a short 29-amino-acid tag on the target protein. The protein can be subsequently purified using the robust and highly specific StrepII affinity marker. (*B*) To purify proteasome complexes, an mNG2[11]-StrepII tag was incorporated into the C terminus of the 20S β4 subunit. (*C*) Purification of the endogenous human proteasome enables the isolation and cryo-EM analysis of different proteasomal complexes, including 20S, PA200–20S, PA28–20S, and 19S–20S. Letters indicate conformational state. (Scale bar, 20 Å.)

We sought to apply the highly resource-efficient CRISPR-Cas9 knock-in pipeline (6, 13), which has recently been demonstrated to work for over ∼1,300 genes in human cells (14), to Expi293 cells for large-scale protein production. For this purpose, we engineered a tandem FP11-StrepII tag (29 amino acids including the linker) (Fig. 1*A*), which is sufficiently small to make use of efficient protocols and minimize structural perturbations on the target protein. FP11s, including GFP[11] (13), mNeonGreen2[11] (15), and sfCherry2[11] (16), are derived by splitting out the last β-strand of fluorescent proteins. Once the tagged protein is expressed, they can spontaneously complement with the corresponding FP1-10 fragment to form a functional fluorescent protein, enabling subsequent sorting of cells containing correct knock-ins by fluorescence-activated cell sorting (FACS). Moreover, we use a transiently expressed FP1-10 fragment, which is depleted over time in cells as observed by the diminishing fluorescence in the cell population after sorting. The proteins purified from the expanded and FP1-10–depleted cells do not contain a bulky, full-sized fluorescent protein that may interfere with structure or function. The entire process, from design of guide RNA (gRNA) and templates to purified protein, can be completed in 3 to 4 wk, with multiple experiments easily performed in parallel.

To demonstrate this approach, we used the human proteasome as a model system. The mammalian proteasome core particle, 20S, is a large, barrel-shaped protein complex composed of four stacked heptameric rings, including two inner β-rings flanked by two outer α-rings that are each formed by seven different α or β subunits (17). The proteolytic sites are located at the N termini of β-subunits (β1, β2, and β5) facing the interior of the proteasome. In each α-ring, the N termini of the α subunits form a gate preventing unregulated protein entry and degradation (18). There are three types of known endogenous proteasomal activators that bind the 20S to open the α-ring gates. One activator is the ATP-dependent 19S complex that recognizes and unfolds ubiquitinated substrates for degradation in an ATP-dependent manner. The function of the other two activators, PA28 and PA200, is ATP-independent. PA28 is up-regulated by interferon-γ during antigen presentation (19, 20) and oxidative stress to degrade damaged and unfolded proteins (21). On the other hand, PA200 is found in the nucleus and is believed to be important for the degradation of histone proteins in DNA repair (22).

To extract the endogenous human proteasome for structural studies, we incorporated a mNG2[11]-StrepII tag onto the C terminus of the 20S β1 (Psmb6), β2 (Psmb7), and β4 (Psmb2) (Fig. 1*B*) subunits in Expi293 cells to create three different cell lines. We targeted multiple subunits because no single gRNA or target is guaranteed to work, so we performed multiple knock-in experiments in parallel to increase our chances of success. mNG2[1-10] was transiently transfected into Expi293 cells. FACS revealed positive knock-in cell populations of 5%, 11%, and 24% for β1, β2, and β4, respectively, reflecting a combined efficiency from knock-in and transient transfection of mNG2[1-10] (*SI Appendix*, Fig. S1). Selecting the β4-StrepII cell population, which had the highest knock-in efficiency, we expanded this cell line and purified the human proteasome by StrepII pull-down.

Affinity purification of human endogenous 20S(β4-StrepII) pulls down multiple proteasomal complexes, including 20S, singly capped with 19S, PA200, and PA28. The purified proteasomal complexes were first visualized by negative-stain EM (*SI Appendix*, Fig. S2), followed by cryo-EM structure determination of singly capped proteasomal complexes of PA28–20S, PA200–20S, and 19S–20S at ∼2.6 to ∼3.2 Å resolution, with and without MG-132 inhibitor (Fig. 1*B* and *C* and *SI Appendix*, Figs. S3 and S4 and Table S1).

The conformational states of the human 19S–20S proteasome have been well-characterized recently (10, 11, 23, 24). The conformations that we captured here (Fig. 1*C*), although all singly capped, are generally consistent with the conformations reported previously. For example, one conformation we captured is the same as the one designated as the ground state (24), in which the axis of the Rpt ring is not aligned with the axis of 20S and the gate in the 20S α-ring is closed. Similarly, the PA200–20S complex is well-resolved and the conformation is consistent with the structure recently reported (12). We therefore did not pursue any further detailed analysis of these complexes.

### Stoichiometry of the Endogenous PA28.

**Fig. 2.**
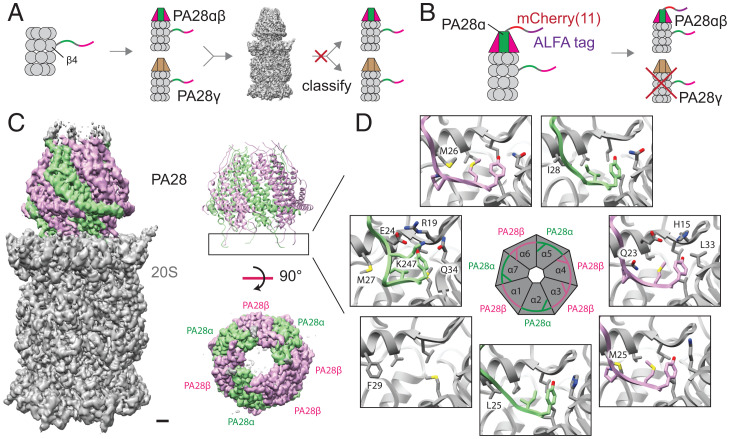
Structure and stoichiometry of the endogenous human PA28–20S proteasomal complex. (*A*) Tagging of the β4 subunit of the 20S proteasome yields both PA28αβ–20S and PA28γ–20S complexes, which cannot be computationally separated during cryo-EM image processing due to their high structural similarity. (*B*) Tagging of the PA28α subunit enables the purification of PA28αβ–20S without PA28γ–20S. (*C*) Cryo-EM map of PA28αβ–20S shows PA28 associating tightly with 20S, resulting in well-defined density for the entire complex. The high-quality density map allows clear assignment of three α and four β subunits in PA28 (*SI Appendix*, Fig. S5). (Scale bar, 10 Å.) (*D*) Six out of seven binding sites on the α-ring of 20S is occupied by the C-terminal tails of PA28 subunits.

Endogenous PA28 subunits are known to form two different heptameric complexes: a heteroheptamer composed of PA28α and PA28β subunits and a homoheptamer composed of PA28γ subunits. Pull-down of 20S(β4-StrepII) is expected to yield both PA28αβ–20S and PA28γ–20S complexes. Indeed, mass spectrometry analysis of the purified proteins reveal all three PA28 subunits to be present in the purified sample (*SI Appendix*, Fig. S5). Furthermore, the resulting cryo-EM map of PA28–20S purified from 20S(β4-StrepII) pull-down shows side-chain density that matches PA28β in some regions while matching PA28γ in other regions (*SI Appendix*, Fig. S5), suggesting that the cryo-EM map is an average of both PA28αβ and PA28γ complexes. However, the high similarity between the two complexes prevented using computational classification to separate them (Fig. 2*A*).

To isolate a homogeneous PA28–20S complex for structural studies, we engineered an sfCherry2[11]-ALFA tag onto the N terminus of the PA28α subunit using the method described above, except here we used a split sfCherry system (16) rather than split mNG2 and ALFA tag instead of StrepII tag, so that we can have orthogonal tags both for sorting and for purification in the same cell line (Fig. 2*B*). The knock-in had an efficiency of ∼7% by FACS. One-step affinity purification using an NbALFA nanobody resin (25) yielded proteasome assemblies capped with PA28, including double-capped proteasomes. Cryo-EM analysis of this sample allowed us to determine structures of single-capped PA28–20S and double-capped PA28–20S–PA28 to ∼2.8 and ∼3.3 Å resolution, respectively (Fig. 2*C* and *SI Appendix*, Figs. S3, S4, and S7*A* and Table S1). Additionally, the tagging of PA28α with sfCherry2[11]-ALFA tag was performed in the cell line that already contained mNG2[11]-StrepII–tagged 20S β4, demonstrating the ability to multiplex orthogonal tags onto different subunits of a larger protein assembly to increase target specificity. The cryo-EM map of PA28αβ–20S purified by ALFA-PA28α pull-down allowed robust modeling of the side-chain densities of PA28αβ, allowing us to distinguish the subunit stoichiometry of the complex.

Previous crystallographic structural analysis of the mouse PA28αβ heteroheptamer found a stoichiometry of four α and three β subunits (26). The protein complex for this previous study was produced by heterologous coexpression of the two subunits, which form a 4α/3β assembly reportedly due to a more thermodynamically stable α–α interface compared to the β–β interface. A more recent cryo-EM study of heterologously expressed human PA28αβ mixed in vitro with bovine 20S also made a similar conclusion (9). Interestingly, our analysis of the endogenous human PA28αβ–20S proteasome found a different subunit stoichiometry of PA28αβ. The high-quality density map of the PA28αβ–20S complex reported here revealed a composition of three α and four β subunits (Fig. 2*C* and *SI Appendix*, Fig. S6 and Table S2), different from the 4α/3β assembly observed previously.

We note that the PA28 in our structure is better-resolved than the previous structure of the human PA28 (9), revealing a stronger engagement between PA28 and 20S in the endogenous complex. Six out of seven C termini of endogenous PA28 engaged in stable interactions with the binding pockets of the 20S α-ring (Fig. 2*D* and *SI Appendix*, Fig. S9). There is one low-occupancy binding site situated between the 20S α1 and α2 subunits where a phenylalanine, 20S α1 F29, may be sterically hindering the binding of the C-terminal tail of PA28β (Fig. 2*D*). The other six unique binding pockets are well-engaged with the corresponding C termini of the PA28 3α/4β assembly. On the other hand, the PA28 4α/3β assembly derived from heterologous coexpression showed only partial association with native 20S when assembled in vitro (9). These results suggest that PA28 in a 3α/4β arrangement may have increased binding affinity to 20S compared to a 4α/3β configuration.

### Protein Loading and Degradation in the PA28–20S Proteasome.

**Fig. 3.**
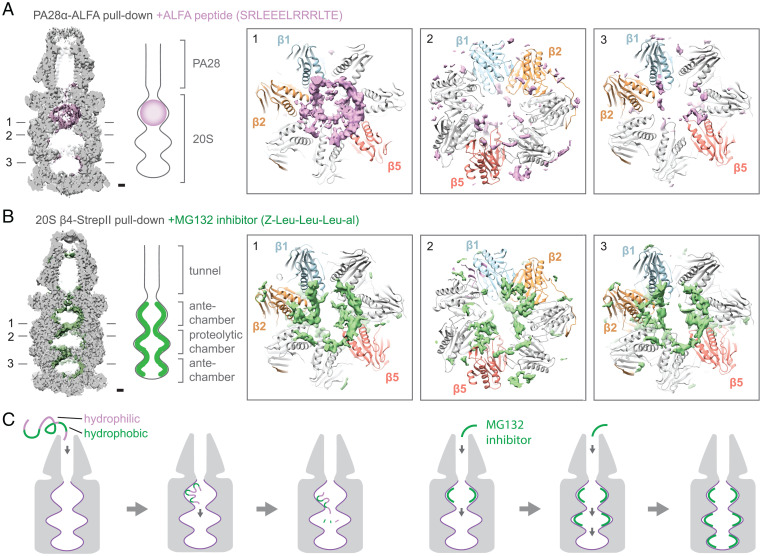
Polypeptide loading and diffusion in the PA28–20S complex. (*A*) Cross-section through the cryo-EM map of PA28–20S shows the presence of additional density, likely ALFA peptide, inside the antechamber of 20S that is adjacent to PA28. The ALFA peptide density is diffusely distributed throughout the PA28-proximal antechamber with higher density along the walls, viewed from the antechamber toward central proteolytic chamber (1). Little density is observed in the proteolytic chamber, viewed from central chamber toward upper antechamber (2) and the PA28-distal antechamber, viewed from bottom antechamber toward central chamber (3). (Scale bar, 10 Å.) (*B*) Addition of MG-132 peptide-like inhibitor shows additional density in all inner chambers of 20S. The density is concentrated along the walls of the inner chambers near the catalytic subunits β1, β2, and β5. (Scale bar, 10 Å.) (*C*) Unfolded polypeptides diffuse quickly through PA28 and bind to the walls of the 20S antechamber. The peptides diffuse along the inner wall into the proteolytic chamber for degradation. Inhibition of proteolysis by MG-132 does not impede the diffusion of molecules inside the 20S proteasome.

Proteasome activators serve to open the 20S gate and facilitate the loading of unfolded polypeptides into the 20S inner chambers for degradation. Passing through the open gate in the α-ring, unfolded polypeptides first enter the antechamber formed between an α- and β-ring and then move into the proteolytic chamber formed between two β-rings for peptide cleavage. Interestingly, the cryo-EM map of PA28αβ–20S purified by ALFA-PA28α pull-down shows additional density inside the 20S antechamber that is proximal to the bound PA28 (Fig. 3*A* and *SI Appendix*, Fig. S7*A*). We suspect that this additional density does not represent endogenous substrates but rather is from ALFA peptide used to elute purified complex. ALFA peptide is present at high concentrations in the elusion buffer and likely enters 20S as substrates through the α-ring gate opened by PA28. Consistent with this argument, cryo-EM analysis of proteasomal complexes purified via StrepII pull-down of 20S(β4-StrepII) did not show similar density inside any map of 20S, PA200–20S, or 19S–20S.

As a short helical peptide, ALFA peptide presumably can interconvert between folded and unfolded states in solution (27), and thus can diffuse as substrates into PA28–20S complex for degradation. One question that arises is whether the observed substrate density represents cleaved or uncleaved ALFA peptides. Since polypeptides are cleaved in the proteolytic chamber, we would expect the cleaved peptides to diffuse from the proteolytic chamber into both antechambers and result in peptide density in both these areas. However, the ALFA peptide density is only observed in the 20S antechamber that is proximal to the bound PA28 (Fig. 3*A*), suggesting that the density could be uncleaved ALFA peptide that has just entered into the 20S proteasome through the PA28 activator rather than cleaved peptides on their way out of the proteasome. This observation also indicates that the diffusion of substrate polypeptides into the PA28–20S proteasome is a relatively fast process compared to the movement of polypeptides from the 20S antechamber into the catalytic chamber. This feature of the PA28–20S proteasome may be important for its role in quickly removing damaged and unfolded proteins from the cellular environment in response to oxidative stress.

Inspection of the ALFA peptide density reveals a nonuniform distribution of density inside the 20S antechamber, with stronger density observed lining the walls of the chamber (Fig. 3*A*). The inner walls of the 20S proteasome are composed of a mix of hydrophilic and hydrophobic regions (*SI Appendix*, Fig. S7*B*), which may serve to bind unfolded polypeptides. The ALFA peptide accumulates in the 20S antechamber and is likely degraded once it enters the proteolytic chamber. To visualize what might happen if proteolysis was blocked, we purified proteasome complexes via 20S(β4-StrepII) by StrepII pull-down in the presence of the peptide-like inhibitor MG-132 and carried out cryo-EM analysis (Fig. 3*B*). Distinct density for MG-132 was observed bound to the catalytic sites of 20S β1, β2, and β5 (*SI Appendix*, Fig. S8), consistent with a previous study of the yeast proteasome (28). Besides the 20S proteolytic chamber, additional density is also observed in both antechambers, indicating that MG-132 can diffuse into all inner chambers of the 20S proteasome. Within each inner chamber, the hydrophobic MG-132 shows a distinct localization to hydrophobic 20S inner wall areas. Furthermore, the distribution of MG-132 shows connected densities leading to the proteolytic sites on β1, β2, and β5 (Fig. 3*B*), which are also observed in the ALFA peptide density and suggest possible routes of peptide movement from the antechamber into the proteolytic chamber. Taken altogether, these data shed light on how unfolded proteins move through the proteasome (Fig. 3*C*). First, unfolded polypeptides enter the 20S antechamber and bind to hydrophobic and hydrophilic regions on the inner chamber wall, which may help to prevent partial refolding of substrates. Consecutive binding and unbinding of the polypeptides to the inner chamber wall allow it to progressively diffuse into the proteolytic chamber for degradation. Inhibition of proteolysis by MG-132 does not inhibit diffusion of peptide-like molecules within the proteasome, indicating that proteolysis is likely decoupled from substrate movement in the proteasome.

## Discussion

Many of the cell’s internal machinery functions as complexes composed of multiple proteins and/or nucleic acid subunits. While traditional methods of protein overexpression have proven useful in the study of numerous protein/nucleic acid assemblies, the study of native biological complexes offers numerous benefits. For large protein assemblies, it is possible to tag a single protein subunit to purify the entire complex without the need to coexpress multiple proteins at the same time and at the correct levels. Native proteins are also likely to be properly folded and functional compared to overexpressed proteins. In this study, we demonstrate tagging of proteins in HEK293 cells. Similar approaches of using CRISPR-Cas gene editing to tag endogenous proteins can likely be applied in many different cell lines including primary and patient-derived disease models. Application of this strategy to disease models could allow us to study the molecular mechanisms of proteins in disease-relevant states, which would help us to better understand how proteins work under disease conditions.

In this study, we report a human PA28 stoichiometry of 3α/4β, which is different from the mouse PA28 stoichiometry of 4α/3β reported previously (24, 26). In the previous study, the authors demonstrated that PA28α and PA28β can also form homoheptameric complexes when the proteins are overexpressed individually. Our attempts at studying the native stoichiometry of free PA28 by native mass spectrometry proved inconclusive, but the data do not discount the possibility that multiple configurations of PA28 may be present in the cell. While our study indicates that PA28(3α/4β) is the preferred configuration that engages with the 20S proteasome, it is possible that PA28 could be present in both 3α/4β and 4α/3β stoichiometries in the cell. However, cryo-EM sample preparation and the image classification procedure are biased against PA28 that engages weakly with 20S, which may exclude complexes with 4α/3β stoichiometry in our final map reconstructions. However, what role PA28 with different stoichiometries play in the cell remain open questions for future study.

Most endogenous proteins exist at much lower concentrations in cells relative to overexpressed proteins, which can make both fluorescence selection and structural analysis of these relatively rare targets challenging. For selection, it has been estimated that ∼50% of the human proteome is expressed above the FACS detection limit using mNG2[11] tagging (14), and signal amplification using our recent tag-assisted split enzyme complementation method (29) could further expand the accessible range. For purification, the recent development of affinity grids offers another potential solution to the study of low-abundance targets (30, 31). Tagging endogenous proteins and purifying them directly on an EM grid can drastically reduce the amount of sample required for structural studies by avoiding intermediate steps (30). Additionally, pulling down proteins directly from lysate can reduce the time the protein spends outside the cell environment, which can help to preserve their native protein–protein interactions and the integrity of delicate protein complexes.

One exciting future direction stemming from the concepts described here is the study of endogenous proteins from animal models. This can allow the structural and biophysical characterization of proteins in their physiological states, which can provide important insights into how proteins function in vivo (32). Efficient strategies to target proteins in native sources will be key to this research area and our study demonstrates important methodologies for achieving this goal. Improved procedures to tag and purify low-expressing proteins and optimized strategies to target native proteins from animal models will be critical areas for future development.

## Methods

### Primers for gRNA Synthesis.

#### ML557.

TAATACGACTCACTATAG

#### ML558.

AAAAAAAGCACCGACTCGGTGC

#### ML611.

AAAAAAAGCACCGACTCGGTGCCACTTTTTCAAGTTGATAACGGACTAGCCTTATTTAAACTTGCTATGCTGTTTCCAGCATAGCTCTTAAAC

#### PSMB2 oligo.

TAATACGACTCACTATAGGATGTTAGGAGCCCTGTTTGGTTTAAGAGCTATGCTGGAA

#### PSMB6 oligo.

TAATACGACTCACTATAGTTATTGCATACTAGAATCCCGTTTAAGAGCTATGCTGGAA

#### PSMB7 oligo.

TAATACGACTCACTATAGGCCACCCACTGATGCCATTCGTTTAAGAGCTATGCTGGAA

#### PSME1 oligo.

TAATACGACTCACTATAGCCCGGTCATGGCCATGCTCAGTTTAAGAGCTATGCTGGAA

### Single-Strand DNA for Homology-Directed Repair (HDR).

#### PSMB2-mNG211-StrepII.

ATTGACAAAAATGGCATCCATGACCTGGATAACATTTCCTTCCCCAAACAGGGCTCCGGCAGCACCGAGCTCAACTTCAAGGAGTGGCAAAAGGCCTTTACCGATATGATGGGGAGTGCGTGGTCCCACCCTCAATTCGAGAAGTAACATCATGTCCTCCCTCCCACTTGCCAGGGAACTTTTTTTTGATGGGCTCCTTT

#### PSMB6-mNG211-StrepII.

CAAGTACTTTTGGGAGACCAGATACCCAAATTCGCCGTTGCCACTTTACCACCCGCCGGCAGCACCGAGCTCAACTTCAAGGAGTGGCAAAAGGCCTTTACCGATATGATGGGGAGTGCGTGGTCCCACCCTCAATTCGAGAAGTGAATACTGGGATTCTAGTATGCAATAAGAGATGCCCTGTACTGATGCAAAATTTA

#### PSMB7-mNG211-StrepII.

ATCACTCCTCTGGAGATTGAGGTGCTGGAAGAAACAGTCCAAACAATGGACACTTCCGGCAGCACCGAGCTCAACTTCAAGGAGTGGCAAAAGGCCTTTACCGATATGATGGGGAGTGCGTGGTCCCACCCTCAATTCGAGAAGTGAATGGCATCAGTGGGTGGCTGGCCGCGGTTCTGGAAGGTGGTGAGCATTGAGGC

#### ALFA-sfCherry211-PSME1.

CCCACTCCACTCCTTGTGCGGCGCTAGGCCCCCCGTCCCGGTCATGCCTTCTAGATTAGAAGAGGAATTGAGACGACGATTGACTGAACCAGGATCTTATACTATCGTTGAACAATACGAGAGAGCTGAGGCAAGACACTCTACAGGTTCTGGCGCCATGCTCAGGGTCCAGCCCGAGGCCCAAGCCAAGGTGAGCGCCG

### gRNA In Vitro-Transcribed (IVT) Template Synthesis.

The IVT template for LMNA gRNA was made by PCR (*SI Appendix*, Fig. S1). The reactions were completed in a 100-μL reaction containing 50 μL 2× Phusion master mix, 2 μL ML557 + 558 mix at 50 μM, 0.5 μL ML611 at 4 μM, 0.5 μL of each gene-specific oligo at 4 μM, and 47 μL diethyl pyrocarbonate (DEPC) H_2_O. PCR program: 95 °C, 3 min; 20× (98 °C, 20 s; 57 °C, 15 s; 72 °C, 5 s); 72 °C, 60 s. The PCR product was purified using a Zymo DNA Clean and Concentrator Kit (Zymo Research D4013) and DNA was eluted in 10 μL. The expected DNA concentration is around 100 ng/µL. DNA was stored at −20 °C until used for gRNA synthesis.

### gRNA Synthesis.

In vitro transcription was carried out using the HiScribe T7 Quick High Yield RNA Synthesis Kit (NEB E2050S) with the addition of RNAsin (Promega N2111). In 20 μL, 300 ng of DNA template, 10 μL NTP buffer mix, 2 μL T7 polymerase, 1 μL RNAsin, and water was added. Addition of RNAsin is important to prevent RNA degradation. The reaction was incubated overnight and the RNA was purified using the RNA Clean and Concentrator Kit (Zymo Research R1017). RNA was eluted in 10 μL with an expected concentration >6 μg/μL. Single-guide RNA (sgRNA) was stored at −80 °C immediately after measuring concentration and diluting to 130 μM.

### Cas9 HDR Knock-Ins.

In a sterile PCR or microcentrifuge tube, 8 μL Cas9 Buffer, 12 μL DEPC H_2_O, and 4 μL sgRNA were mixed and incubated at 70 °C for 5 min to refold the gRNA. During this step, 10-μL aliquots of purified Cas9 at 40 μM were thawed on ice and once thawed were slowly added to the diluted sgRNA in Cas9 buffer and incubated at 37 °C for 10 min for ribonucleoprotein complex formation. Six microliters of HDR template donor was added to the ribonucleoprotein mix, and all samples were kept on ice until ready for nucleofection. For efficient recovery post-knock-in (KI), a six-well plate with 2 mL of Expi293F expression media (Thermo Fisher) per well was incubated at 37 °C. An appropriate amount of supplemented Lonza Amaxa solution corresponding to the number of KIs to be performed was prepared at room temperature in the cell culture hood. For each sample, 65.6 μL of SF solution (Lonza Group Ltd.) and 14.4 μL of supplement was added to an Eppendorf tube for a total of 80 μL per KI. Lonza nucleofector instruments/computers were then turned on and kept ready for nucleofection. The Amaxa solution is toxic to cells, so it is important to make sure everything is ready to go beforehand.

Cells (800,000 per KI) were harvested by centrifugation at 500 × *g* for 3 min.Supernatant was removed and cells were resuspended in 1 mL of phosphate-buffered saline to wash. The cells were centrifuged again at 500 × *g* for 3 min. PCR tubes containing ribonucleoproteins were brought into the tissue culture hood. Cells were resuspended in 80 μL of supplemented Amaxa solution and the cell resuspension was added to the 40 μL of preformed ribonucleoprotein + HDR template mix. The 120-μL mixture was pipetted into the bottom of the nucleofection plate, avoiding air bubbles. The nucleofection was carried out in 100-μL cuvettes on a Lonza Nucleofector X Unit (Lonza AAF-1002X) attached to Lonza 4D Nucleofector Core Unit (Lonza AAF-1002B). Cells were nucleofected using the FS-100 program and immediately recovered using 400 μL of media from the prewarmed six-well plate and transferred to the corresponding well. Because the Amaxa solution is toxic to the cells, it is important to perform these steps quickly. The cells were incubated at 37 °C and 8% CO_2_.

Cells were monitored over 5 to 7 d. Many cells will look unhealthy and die, and growth rate will slow dramatically. Cells will begin to recover after 5 d. Once cells expanded to the point where they began to detach from the well bottom, the plate was transferred to a shaker operating at 120 rpm and incubated at 37 °C and 8% CO_2_. The next day, the cells were transferred to a 125-mL vented flat-bottom Erlenmeyer flask containing 20 mL Expi293F media and incubated at 37 °C and 8% CO_2_ with shaking at 120 rpm until the cell density reached 1 million/mL.

### Transfection of mNG2 (1-10).

To prepare the transfection mixture, 20 μg of pSFFV-mNG2 (1-10) plasmid was added to 1 mL of Opti-MEM (Gibco) in one tube and 53 μL of ExpiFectamine 293 (Gibco) was added to 1 mL of Opti-MEM (Gibco) in a separate tube. The mixture was incubated at room temperature for 5 min. The Opti-MEM containing Expifectamine 293 was then added to the solution containing DNA, inverted multiple times to mix, and then incubated at room temperature for 25 min. The transfection mixture was then added to 20 million Expi293F cells in 20 mL of media in a 125-mL vented flat-bottom Erlenmeyer flask. The cells were incubated at 37 °C for 2 d with shaking at 120 rpm. If the knock-in was successful, some cells will show green fluorescence the next day, which will get brighter on day 2.

### Flow Cytometry Analysis and FACS.

FACS sorting and flow cytometry were performed on a BD FACSAria II in the Laboratory for Cell Analysis at the University of California, San Francisco (UCSF). mNG2 signal was measured with the 488-nm laser and 530/30 bandpass filter. One million cells per sample were sorted into Eppendorf tubes and then plated in a six-well plate. The six-well plate was incubated at 37 °C and 8% CO_2_ for 2 d with shaking at 120 rpm. Cells were transferred to 20 mL of Expi293F media in a 125-mL vented Erlenmeyer flask. When the cell density reached 2 × 10^6^ per mL, cells were transferred to 100 mL of Expi293F media in a 500-mL vented Erlenmeyer flask. When cells reached a density of 2 × 10^6^ per mL, cells were harvested and frozen in aliquots.

### Complementary DNA (cDNA) Analysis.

Total RNA was extracted from 1 million cells using the Monarch Total RNA Miniprep Kit (NEB T2010S). cDNA was prepared from 1 µg of extracted RNA using LunaScript RT SuperMix Kit (NEB E3010). No Template and No Reverse Transcriptase controls (NTC and NRT) were performed in parallel to cDNA preparations. cDNA was analyzed in a 2% agarose gel. Sequencing confirmation of amplicons was completed by Elimbio.

### Cell Culture and Protein Purification.

Expi293F cell lines were grown in 100 mL Expi293 expression medium (Thermo Fisher) in 500-mL flat-bottom Erlenmeyer flasks at 37 °C, 8% CO_2_, and shaking at 120 rpm. When cells reached a density of 3 million cells per mL, cells were harvested by centrifugation at 1,000 × *g* for 5 min. Cells were resuspended in 20 mL Buffer A (50 mM Hepes, 150 mM NaCl, and 1 mM dithiothreitol, pH 7.5) supplemented with SigmaFast protease inhibitor (Sigma). Cells were lysed by sonication and insoluble debris were removed by ultracentrifugation at 100,000 × *g* for 20 min. For StrepII-tagged proteins, the supernatant was passed through a column containing 0.2 mL Strep-Tactin XT Sepharose resin (Cytiva) by gravity flow. The resin was washed with 2 mL Buffer A and the bound proteins were eluted with 1 mL Buffer A supplemented with 50 mM biotin (final concentration). For ALFA-tagged proteins, the supernatant was passed through a column containing 0.2 mL ALFA Selector PE resin (NanoTag Biotechnologies) by gravity flow. The resin was washed with 2 mL Buffer A and the bound proteins were eluted at room temperature with 1 mL Buffer A supplemented with 200 μM ALFA peptide (final concentration). To acquire 19S-bound proteasome complexes, 1 mM AMPPNP (Sigma) and 2 mM MgCl were included throughout the purification. To acquire MG-132 bound proteasomal complexes, cells were treated with 5 μM MG-132 for 15 min prior to harvesting and MG-132 was maintained throughout the purification, along with 1 mM ATP. The eluted proteins were concentrated in a 100 K centrifugal concentrating device.

### Negative-Stain Transmission Electron Microscopy Imaging.

Negative-stain EM grids were prepared following established protocol (33). Specifically, 3.5 μL of purified proteasome complexes at 0.02 mg/mL was pipetted onto a continuous carbon-coated grid and incubated at room temperature for 60 s. The sample was blotted using filter paper, washed with 3.5 μL of water, and blotted again with filter paper. Immediately, 3.5 μL of 2% (wt/vol) uranyl formate was pipetted onto the grid and blotted away with filter paper. This treatment with uranyl formate stain was repeated two more times, except with an addition of a 30-s wait between stain application and blotting during the last cycle. The grid was left to dry at room temperature for 2 min. The grids were imaged in a FEI Tecnai T12 fitted with a Gatan US4000 charge-coupled device camera. The images were processed using CryoSPARC (34).

### Cryo-EM Imaging and Data Processing.

To prepare sample grids, 3.5 μL of purified PSMB2-mNG2 (11)-strepII and mCherry (11)-ALFA-PSME1 proteasome complexes at 2 mg/mL was applied to a Quantifoil Au 300 mesh 1.2/1.3 grid (glow-discharged in a Pelco easi-glow at 15 mA for 30 s) in a Vitrobot Mark IV (Thermo), blotted for 8 s (4 C, 100% humidity, blot force 0) using Whatman 1 filter paper, and plunge-frozen in liquid ethane. The sample grids were imaged in a FEI Polara equipped with field emission source and operated at 300 kV. Cryo-EM data acquisition was performed by using SerialEM by single-shot acquisition without image shift (35). Images were recorded using a K2 Summit electron detector operated in superresolution mode using eight electrons per pixel per second at 1.22 Å per physical pixel. The images were corrected for specimen drift using MotionCor2 (36). Non-dose-weighted sums were used for contrast transfer function (CTF) determination using gCTF, and dose-weighted sums were used for the rest of the single-particle cryo-EM image processing. For PSMB2-mNG2 (11)-strepII with MG132, 3.5 μL of sample at 2 mg/mL was applied to a Quantifoil Cu 300 mesh 2/2 grid (glow-discharged in a Pelco easi-glow at 15 mA for 30 s) in a Vitrobot Mark IV, blotted for 10 s (4 C, 100% humidity, blot force 0) using Whatman 1 filter paper, and plunge frozen in liquid ethane. The grids were imaged in an FEI/Thermo Fisher Titan Krios at 300 kV. Cryo-EM data acquisition was performed using SerialEM by image shift (four shots per hole) with beam-tilt compensation (maximum shift ∼0.7 μm). Images were recorded using a Gatan K3 detector in superresolution mode using 25 electrons per pixel per second at 1.06 Å per physical pixel. Motion correction was completed in cryoSPARC using Patch motion correction using default parameters. Particle picking, classification, and refinement were processed in CryoSPARC (34) and *cis*TEM (37). Particle picking was done initially with the blob picker function in cryoSPARC using a blob of 100-Å diameter. From two-dimensional (2D) classification of the resulting particle images (20 iterations), a 2D class of the 20S proteasome (“side” view) was then used to pick particles again in cryoSPARC with a mask diameter of 180 Å. Two-dimensional classification of the extracted images using 100 classes (20 iterations) was used to remove junk/nonproteasome particles in cryoSPARC. The proteasome particle images were aligned to an existing 20S map in cryoSPARC homogeneous refinement (initial low-pass-filtered to 20 Å) to generate an averaged map, which was used as a reference for three-dimensional (3D) classification. The images were subjected to 3D classification using cryoSPARC multirefinement with 10 classes (20-Å resolution limit) to separate the different proteasome complexes. Individual classes were refined with cryoSPARC homogeneous refinement to high-resolution (initial low-pass-filtered to 20 Å). For PSMB2-mNG2 (11)-strepII and mCherry (11)-ALFA-PSME1 samples, maps were further refined in *cis*TEM using estimated molecular weights of 760, 200, and 200 kDa for 20S, PA200, and PA28 complexes (initial low-pass-filtered to 20 Å). For PSMB2-mNG2 (11)-strepII with MG132, the 19S-20S classes were grouped and subjected to another round of 3D classification using cryoSPARC multirefinement (20-Å resolution limit) with eight classes to separate the different conformations. The resulting classes were refined to high resolution using cryoSPARC homogeneous refinement (initial low-pass-filtered to 20 Å). Maps were filtered by local resolution in cryoSPARC. Atomic models were built into the cryo-EM density maps using Coot (38, 39) and Phenix (40, 41) with Torsion, Planar Peptide, Trans Peptide, and Ramachandran restraints turned on. Initial models used were Protein Data Bank (PDB) ID codes 6RGQ (20S), 6KWY (PA200), 7DR6 (PA28), and 5VFQ (19S). One round of Phenix refinement was used at the end to optimize global geometry with a maximum resolution of 3.5 Å. The resulting cryo-EM maps and models were visualized using UCSF Chimera (42).

## Supplementary Material

Supplementary File

## Data Availability

Cryo-EM density maps and atomic models reported in this study have been deposited in the Electron Microscopy Data Bank (EMDB) and Protein Data Bank (PDB) under the accession codes EMD-24275 and PDB 7NAN (20S), EMD-24276 and PDB 7NAO (PA28-20S-3a4b) and PDB 8CXB (PA28-20S-4a3b), EMD-24277 and PDB 7NAP (PA28-20S-PA28), and EMD-24278 and PDB 7NAQ (PA200-20S). Maps and models for proteasomal complexes treated with MG-132 are deposited under accession codes EMD-27013 and PDB 8CVR (20S), EMD-27015 and PDB 8CVS (PA200-20S), EMD-27014 (PA28-20S), EMD-27016 (19S-20S, SA/EA), EMD-27017 (19S-20S, SD1), EMD-27018 and PDB 8CVT (19S-20S, SD2/D2), and EMD-27019 (19S-20S, SD3/C2). All other study data are included in the article and/or *SI Appendix*.
