# Supplementary material for: Structural insights into the human PA28–20S proteasome enabled by efficient tagging and purification of endogenous proteins
Source: Proc Natl Acad Sci U S A. 2022 Jul 19;119(33):e2207200119. doi: 10.1073/pnas.2207200119 (PMC9388094; doi:10.1073/pnas.2207200119)
Supplement: Supplementary File [file pnas.2207200119.sapp.pdf]

Structural insights into human PA28-20S proteasome enabled by efficient tagging and purification of endogenous proteins

Jianhua Zhao[1,#,*,$], Suraj Makhija[2,&,*], Chenyu Zhou[#], Hanxiao Zhang[#], Yongqiang Wang[5], Monita Muralidharan[6], Bo Huang[3,4,$], Yifan Cheng[1,5,$]

[1] Department of Biochemistry and Biophysics, University of California San Francisco. San Francisco, CA 94143, USA
[2] UC Berkeley – UCSF Joint Graduate Program in Bioengineering, University of California, San Francisco, CA 94143, USA
[3] Department of Pharmaceutical Chemistry, University of California San Francisco, San Francisco, CA 94143, USA
[4] Chan Zuckerberg Biohub, San Francisco, CA 94158, USA
[5] Howard Hughes Medical Institute, University of California San Francisco, San Francisco, CA, 94143, USA
[6] Department of Cellular Molecular Pharmacology, University of California San Francisco, San Francisco, CA 94143, USA

* These authors contributed equally

[$] Correspondence: jzhao@sbpdiscovery.org, Bo.Huang@ucsf.edu and Yifan.Cheng@ucsf.edu

Supplementary Figures

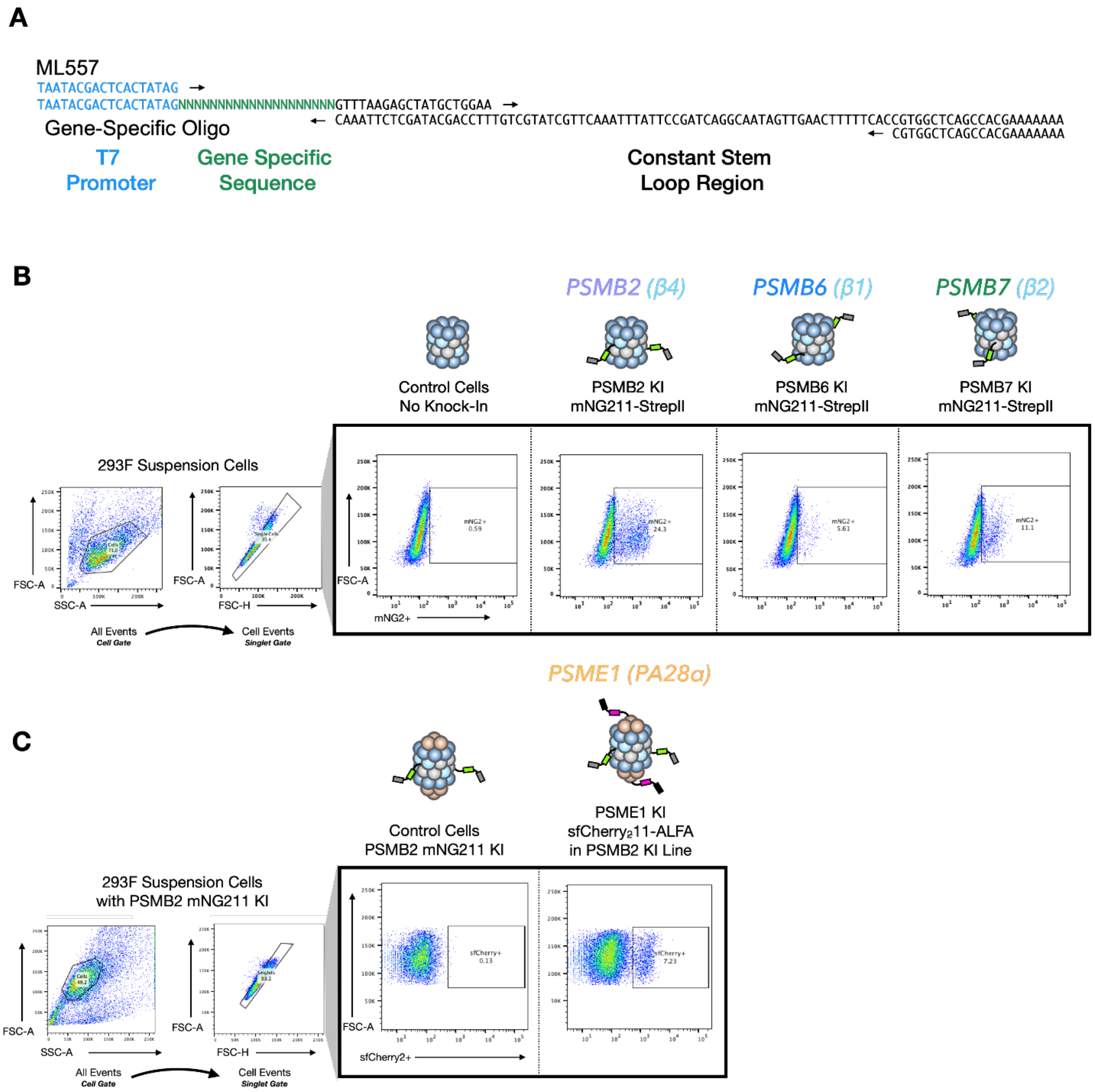

Supplementary Figure 1. gRNA IVT Template Design and FACS Sorting of KIs. (**A**) PCR schematic for the IVT template to make Cas9 gRNA. The gene specific primers (provided in the Methods section) anneal to ML611 containing the constant stem loop region. ML557 and ML558 are used to amplify the gene-specific template created by the gene-specific oligo and ML611. (**B**) FACS sorting of PSMB knock-in Expi293F cells. Shown are control cells used to set gating and PSMB2, PSMB6, and PSMB7 KIs containing the mNG211-StrepII donor. Cells in the gate were collected for downstream structural studies. (**C**) FACS sorting of PSME1 knock-in. This knock-in was performed in the sorted PSMB2 KI cell line. Shown are control cells used to set gating and PSME1 KI containing the sfCherry$_2$11-StrepII donor. Cells in the gate were collected for downstream structural studies.

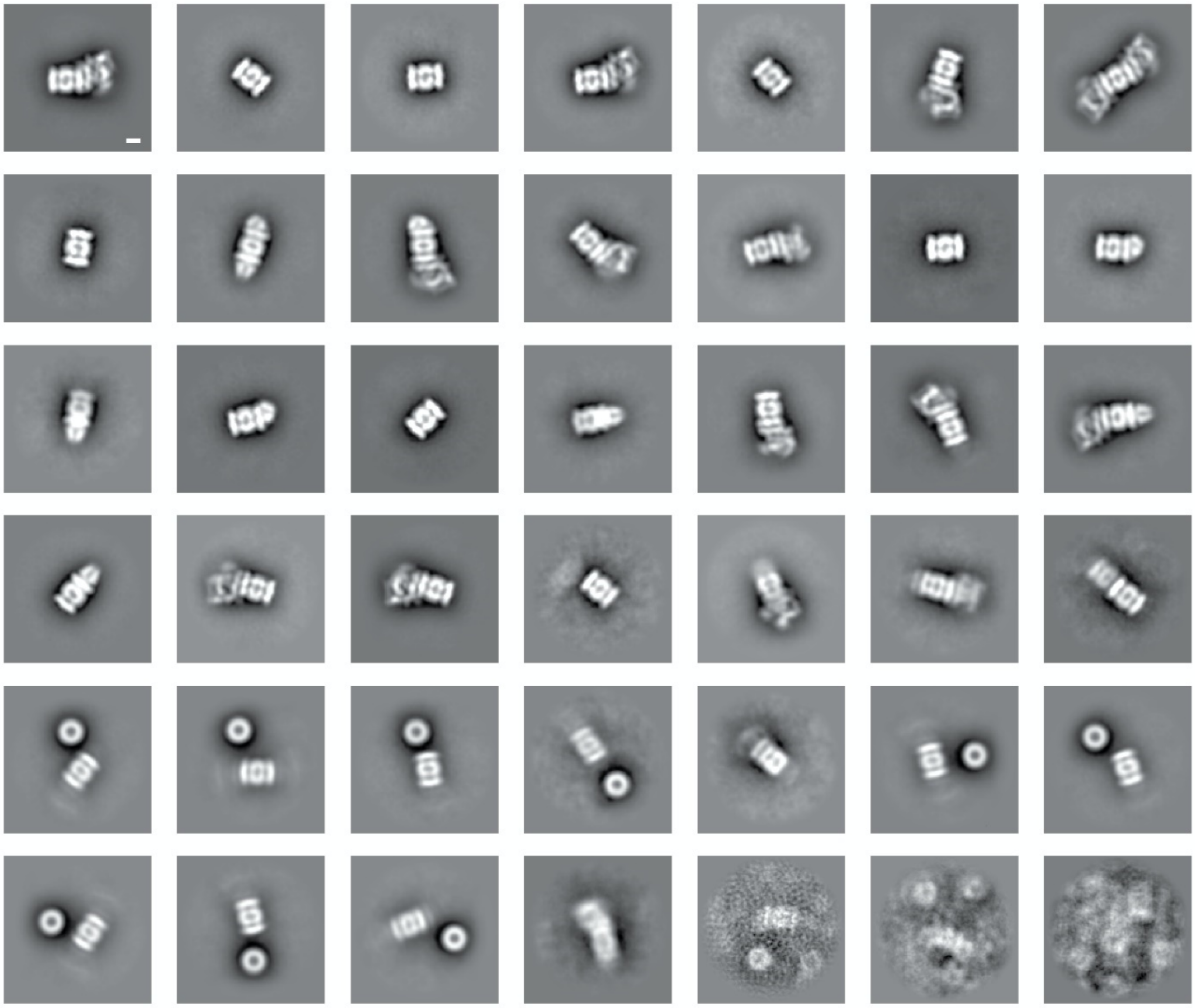

Supplementary Figure 2. Negative stain EM of β4-tagged proteasomal complexes. 2D classification of the images show a diverse population of different proteasomal complexes formed between 20S, 19S, PA28, and PA200. Scale bar, 50 Å.

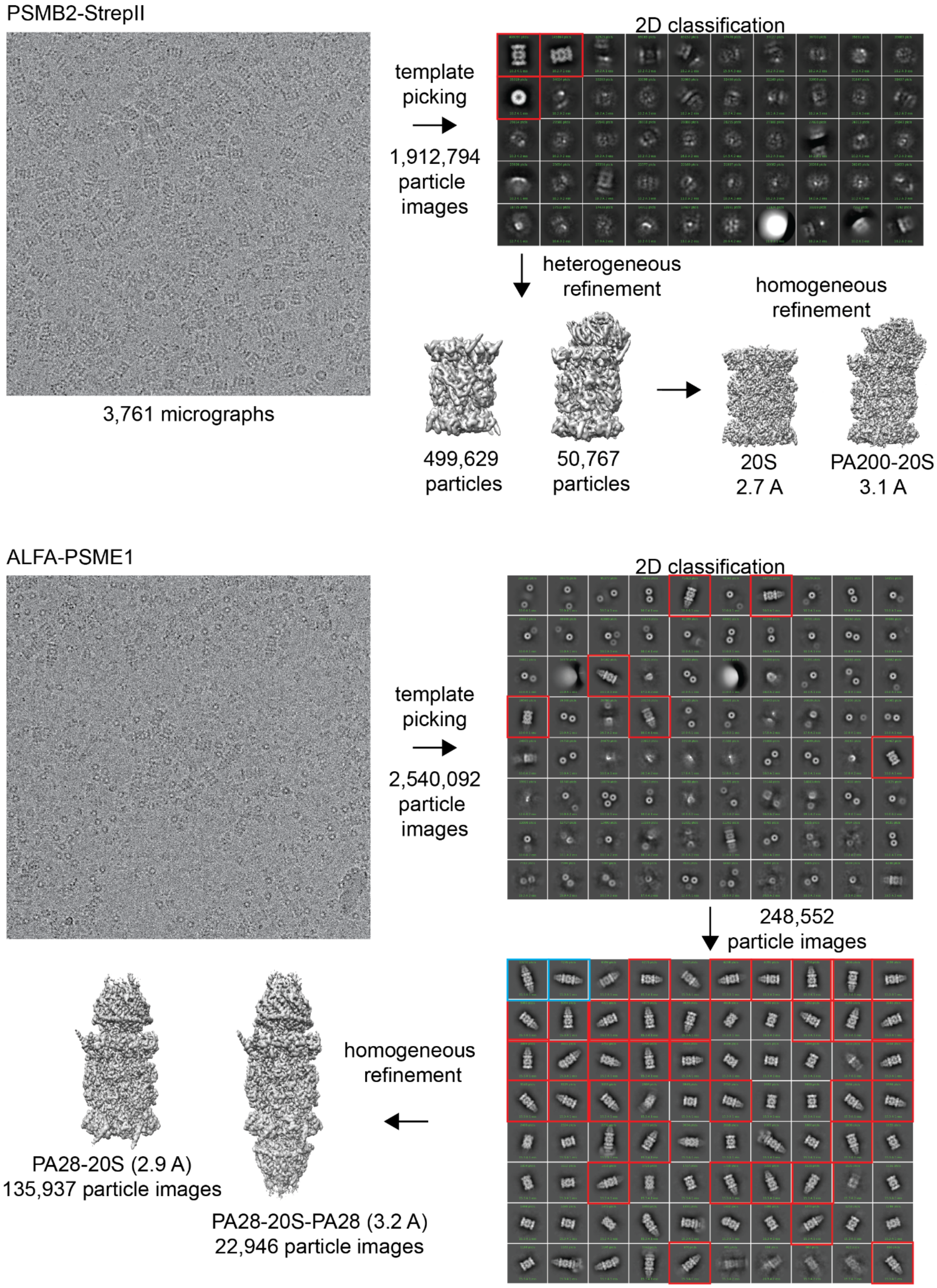
PSMB2-StrepII
3,761 micrographs
template picking
1,912,794 particle images
2D classification
heterogeneous refinement
homogeneous refinement
499,629 particles
50,767 particles
20S
2.7 A
PA200-20S
3.1 A
ALFA-PSME1
template picking
2,540,092 particle images
2D classification
248,552 particle images
homogeneous refinement
PA28-20S (2.9 A)
135,937 particle images
PA28-20S-PA28 (3.2 A)
22,946 particle images

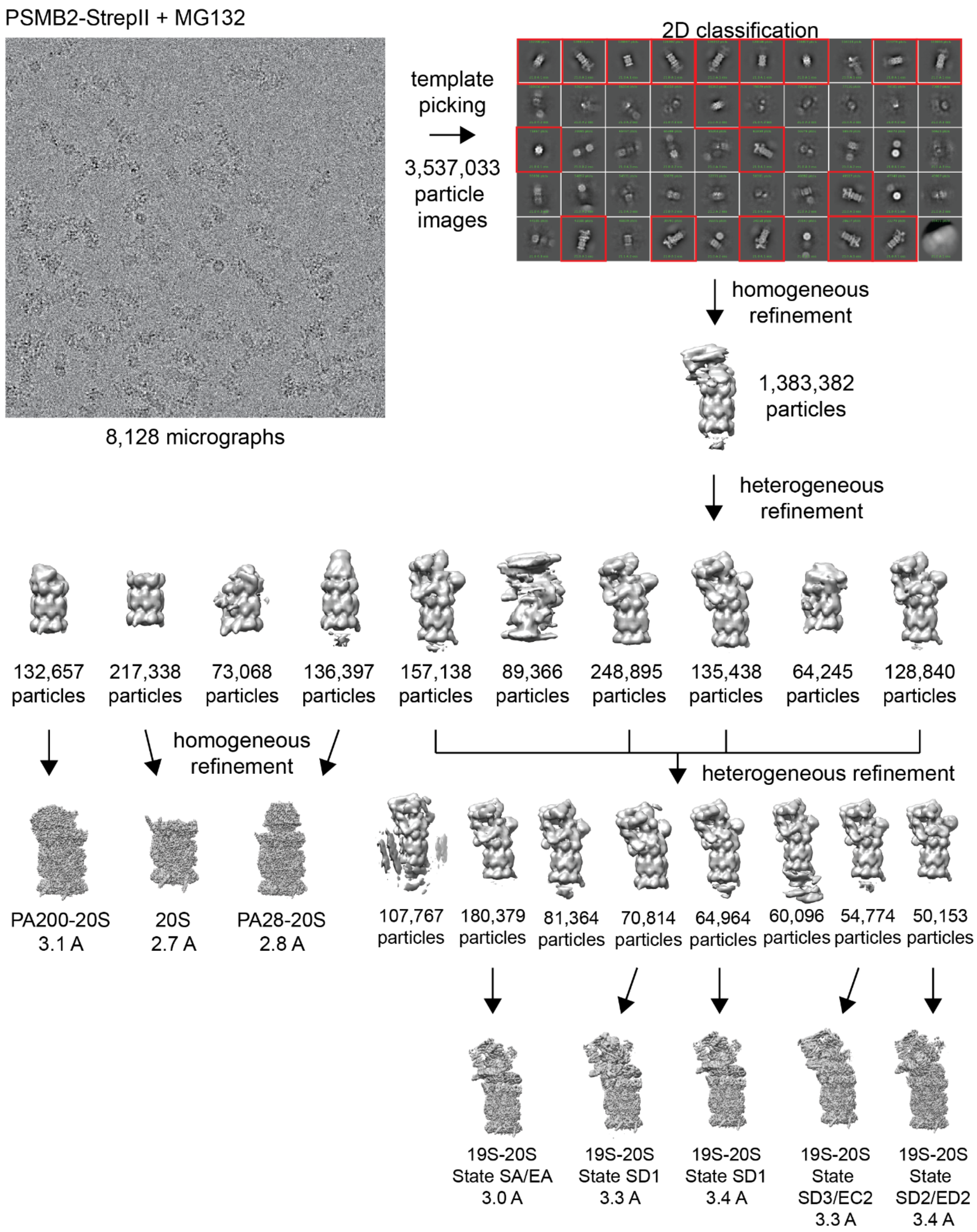

Supplementary Figure 3. Cryo-EM image processing workflow. 2D and 3D classification for PSMB2-StrepII, ALFA-PSME1, and PSMB2-StrepII + MG132 samples.

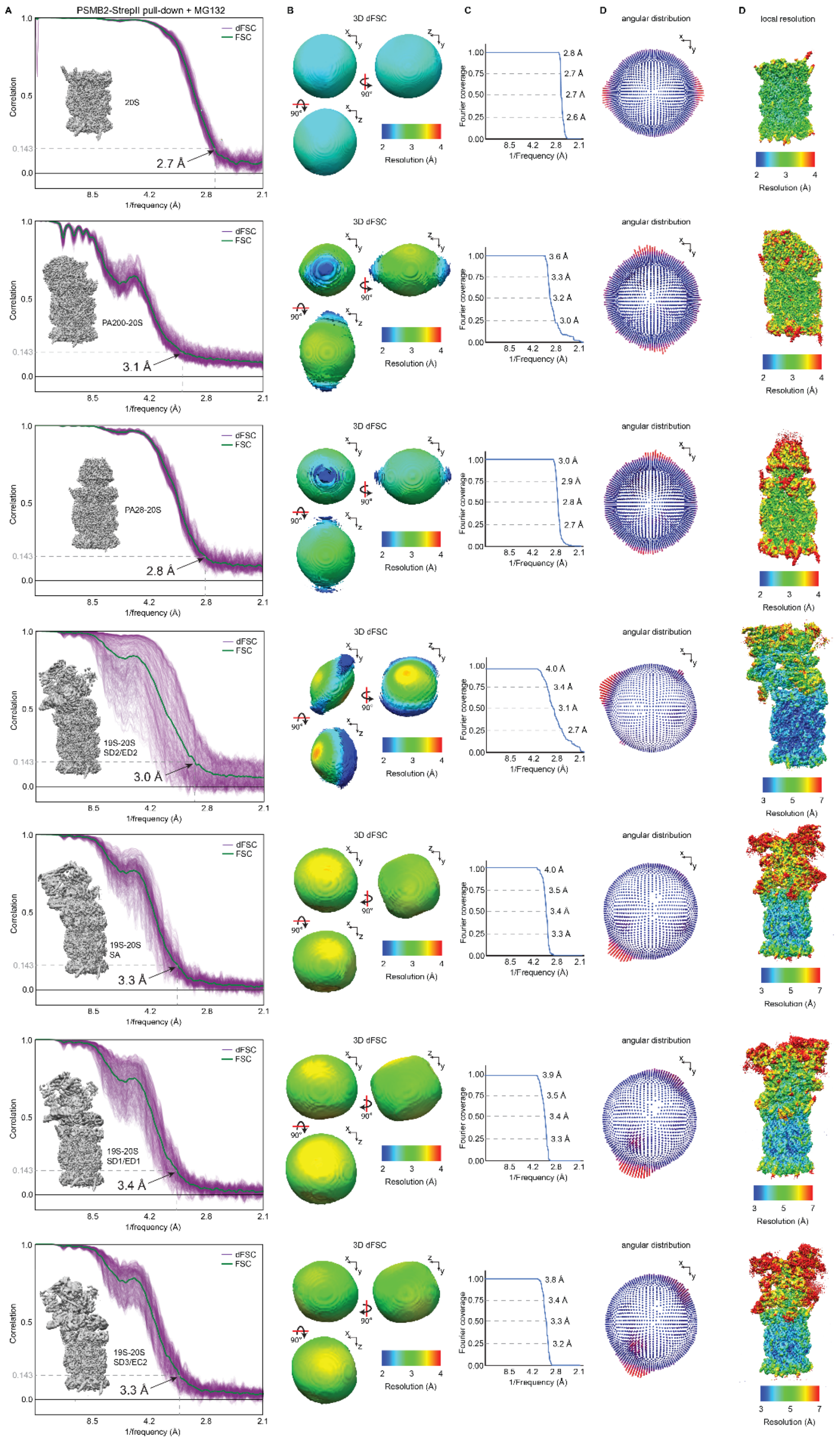

A
PSMB2-StrepII pull-down + MG132
B
3D dFSC
C
D
angular distribution
D
local resolution
Correlation
1/frequency (Å)
dFSC
FSC
20S
2.7 Å
PA200-20S
3.1 Å
PA28-20S
2.8 Å
19S-20S SD2/ED2
3.0 Å
19S-20S SA
3.3 Å
19S-20S SD1/ED1
3.4 Å
19S-20S SD3/EC2
3.3 Å
Resolution (Å)
Fourier coverage
1/Frequency (Å)

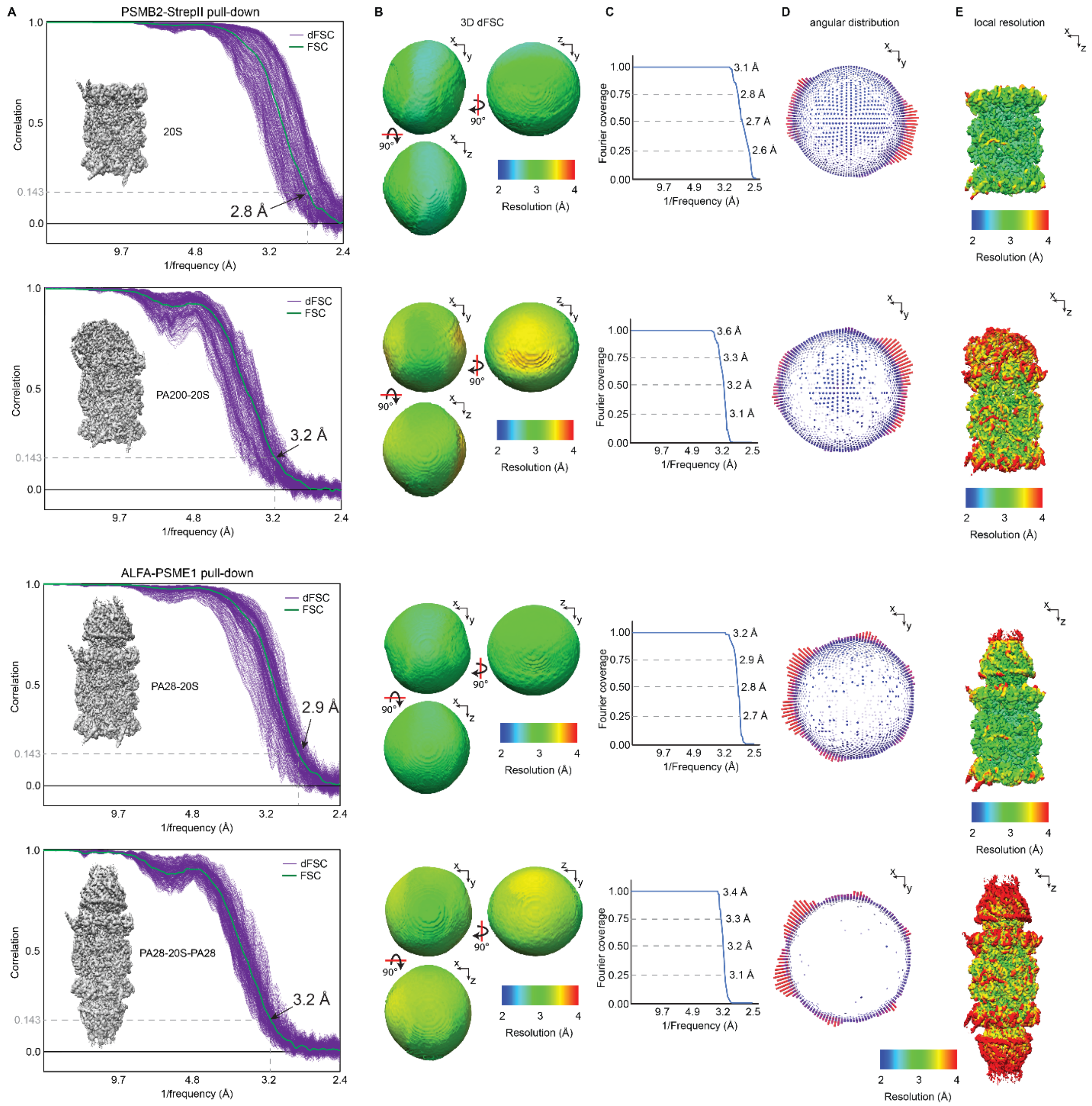

Supplementary Figure 4. Cryo-EM map statistics. (**A**) Fourier shell correlation (FSC) and 1D directional FSC (dFSC) plots. (**B**) Visualization of 3D dFSC. (**C**) Plots showing coverage of Fourier space. (**D**) Plots showing distribution of viewing angles from final refined datasets. (**E**) Corresponding local resolution map with color scale.

Gene	Seq. coverage (%)	Gene	Seq. coverage (%)
PSMB5	75.3	PSMD11	70.1
PSMA7	74.6	PSMD13	76.9
PSMA5	87.1	PSMC3	67.2
PSMB1	66.4	PSMC5	70
PSMA6	91.9	PSMD12	55.3
PSMA1	77.9	PSME3	64.6
PSMA4	72.4	PSMD4	52.8
PSMB6	77.4	PSMC1	66.6
PSMB7	71.8	PSMC6	65
PSMB4	75	PSMD7	58
PSMD1	59.7	PSMC4	73.9
PSMD2	76	PSME1	67.5
PSMA2	80.8	PSME2	69
PSMA3	80	HSPA1B;HSPA1A	61.3
PSMB2	88.6	PSMD14	69.4
PSME4	52.6	ADRM1	53.1
PSMB3	70.2	RPS27A;UBA52	55.8
PSMC2	71.4	TXNL1	66.1
PSMD6	68.9	PSMD8	46.9

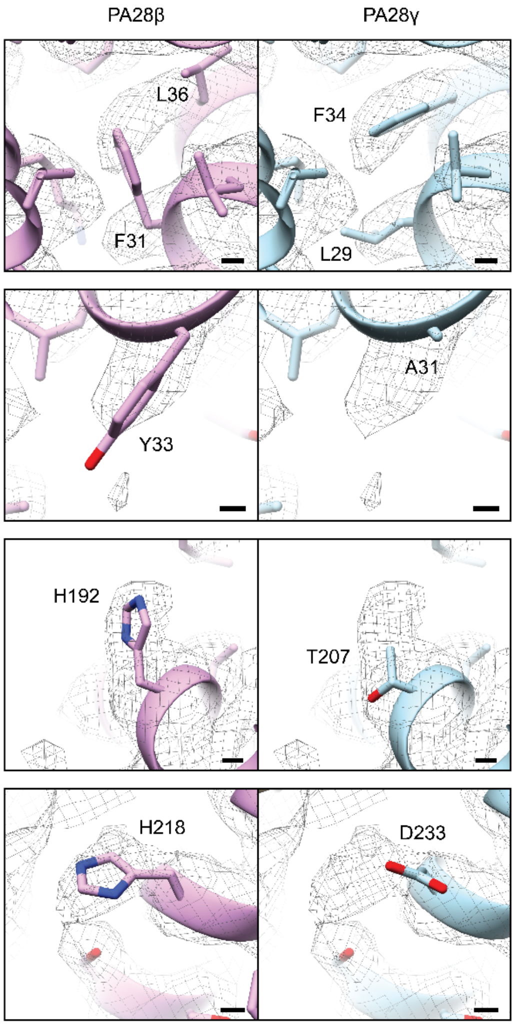

Supplementary Figure 5. Mass spectrometry (left) of 20S(β4-StrepII) pull-down shows co-purification with PA28α (PSME1), PA28β (PSME2), and PA28γ (PSME3). Cryo-EM density map of PA28-20S(β4-StrepII) was modeled with both PA28αβ and PA28γ (right). The density fits PA28γ sidechains in some regions (F34, L29) while PA28β sidechains are better fit in other regions (Y33, H192, H218), indicating that the map is an average of PA28αβ and PA28γ.

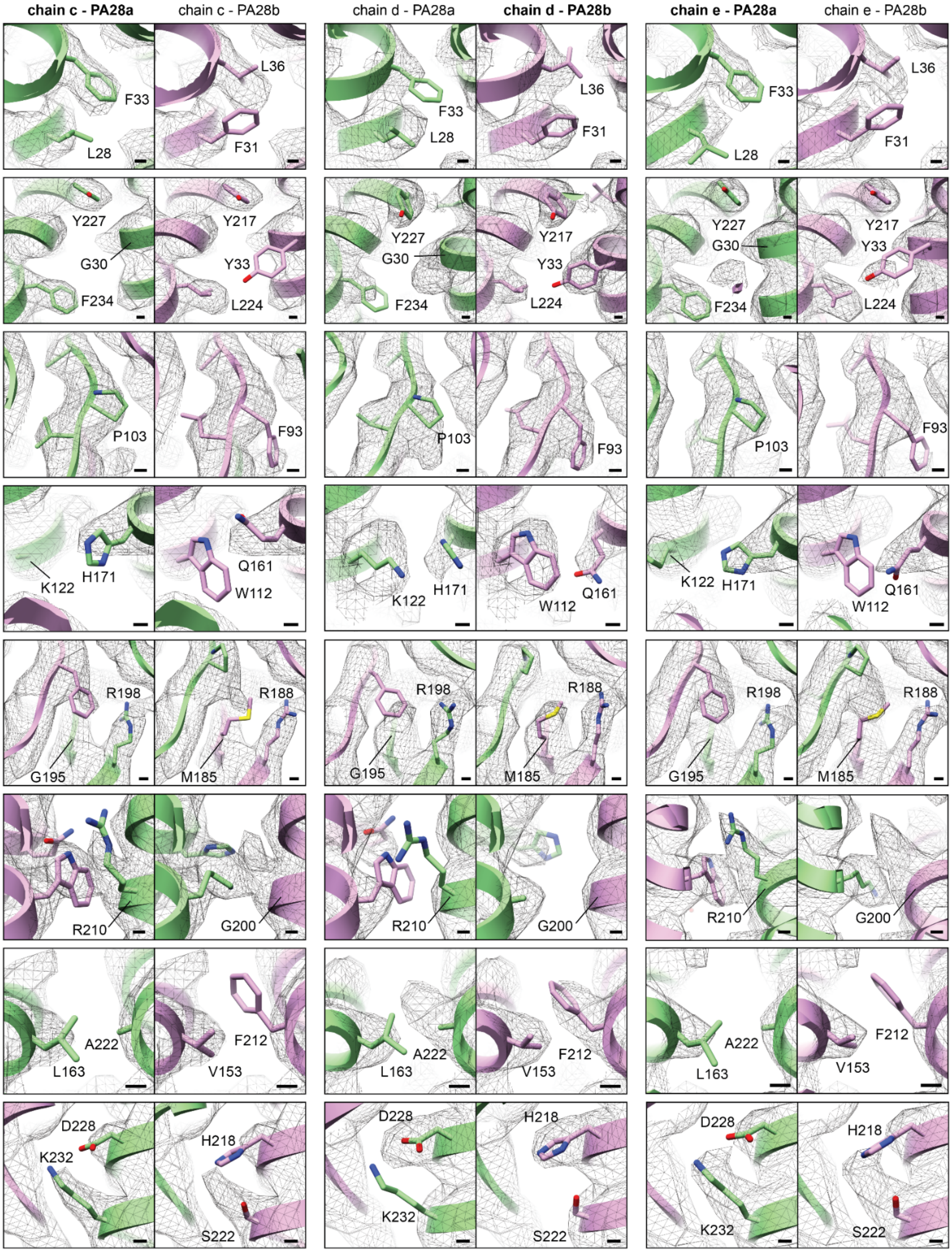

chain c - PA28a
chain c - PA28b
chain d - PA28a
chain d - PA28b
chain e - PA28a
chain e - PA28b
F33
L28
L36
F31
F33
L28
L36
F31
F33
L28
L36
F31
Y227
G30
F234
Y217
Y33
L224
Y227
G30
F234
Y217
Y33
L224
Y227
G30
F234
Y217
Y33
L224
P103
F93
P103
F93
P103
F93
K122
H171
Q161
W112
K122
H171
W112
Q161
K122
H171
W112
Q161
R198
G195
R188
M185
R198
G195
R188
M185
R198
G195
R188
M185
R210
G200
R210
G200
R210
G200
A222
L163
F212
V153
A222
L163
F212
V153
A222
L163
F212
V153
D228
K232
H218
S222
D228
K232
H218
S222
D228
K232
H218
S222

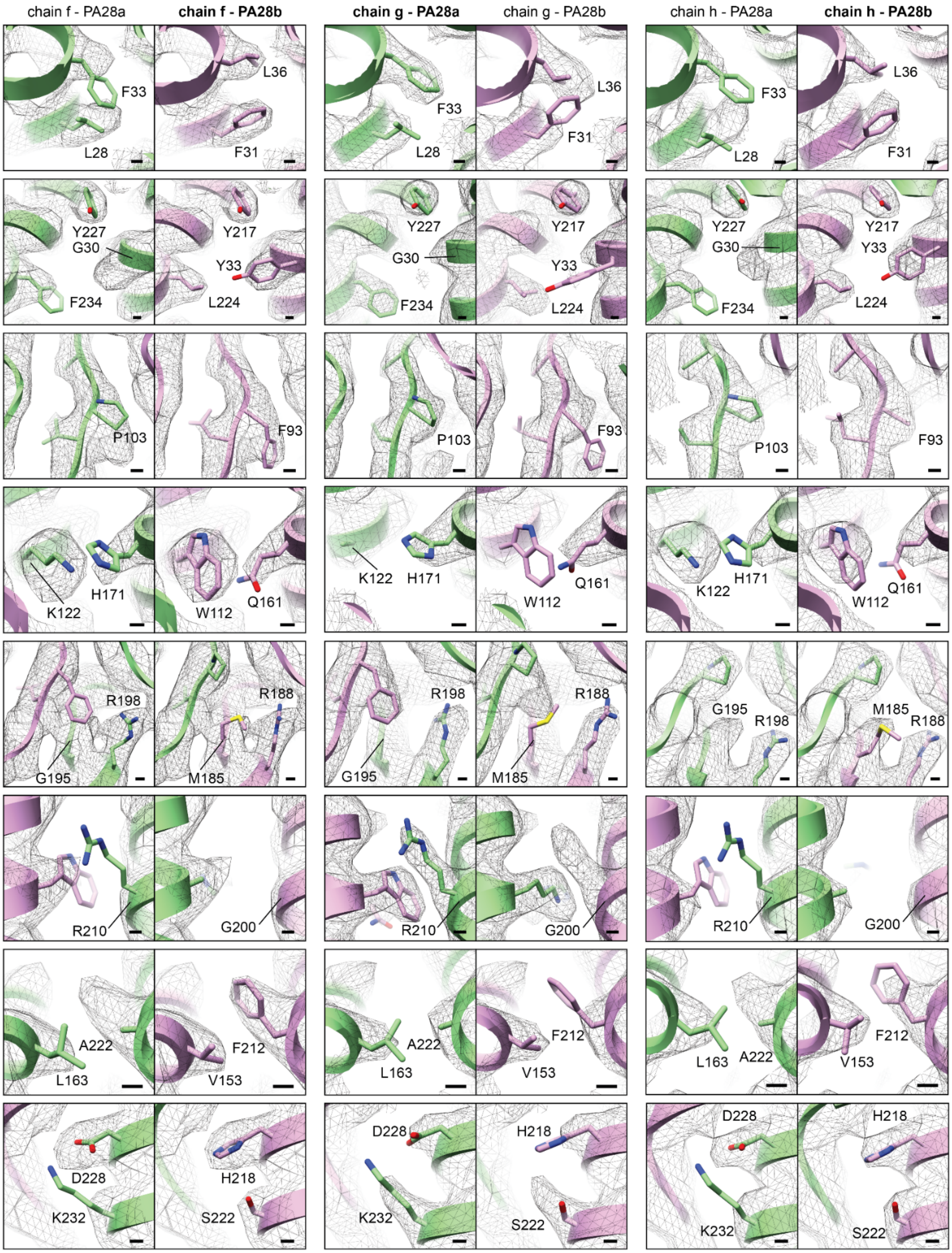

chain f - PA28a
chain f - PA28b
chain g - PA28a
chain g - PA28b
chain h - PA28a
chain h - PA28b
F33
L28
L36
F31
Y227
G30
F234
Y217
Y33
L224
P103
F93
K122
H171
W112
Q161
R198
G195
R188
M185
R210
G200
A222
L163
F212
V153
D228
K232
H218
S222

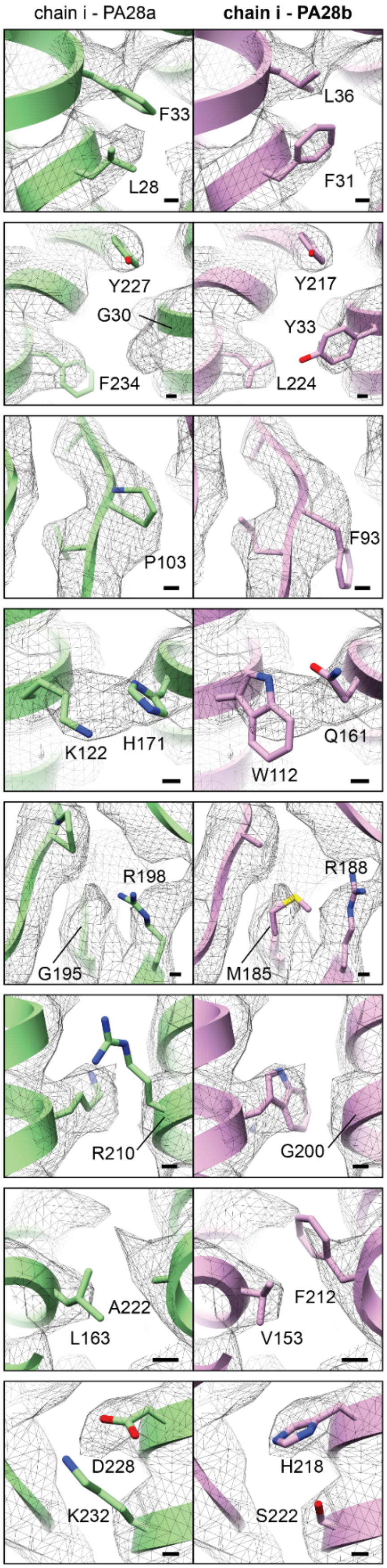

Supplementary Figure 6. PA28 model fit to map density. For each chain in PA28, PA28a (PSME1) and PA28b (PSME2) were modeled into the cryo-EM density map of single capped PA28-20S proteasome. Key differences in the model-to-map fit are highlighted and support a stoichiometry of {a,b,a,b,a,b,b} for chains {c,d,e,f,g,h,i}, respectively. Scale bar, 1 Angstrom.

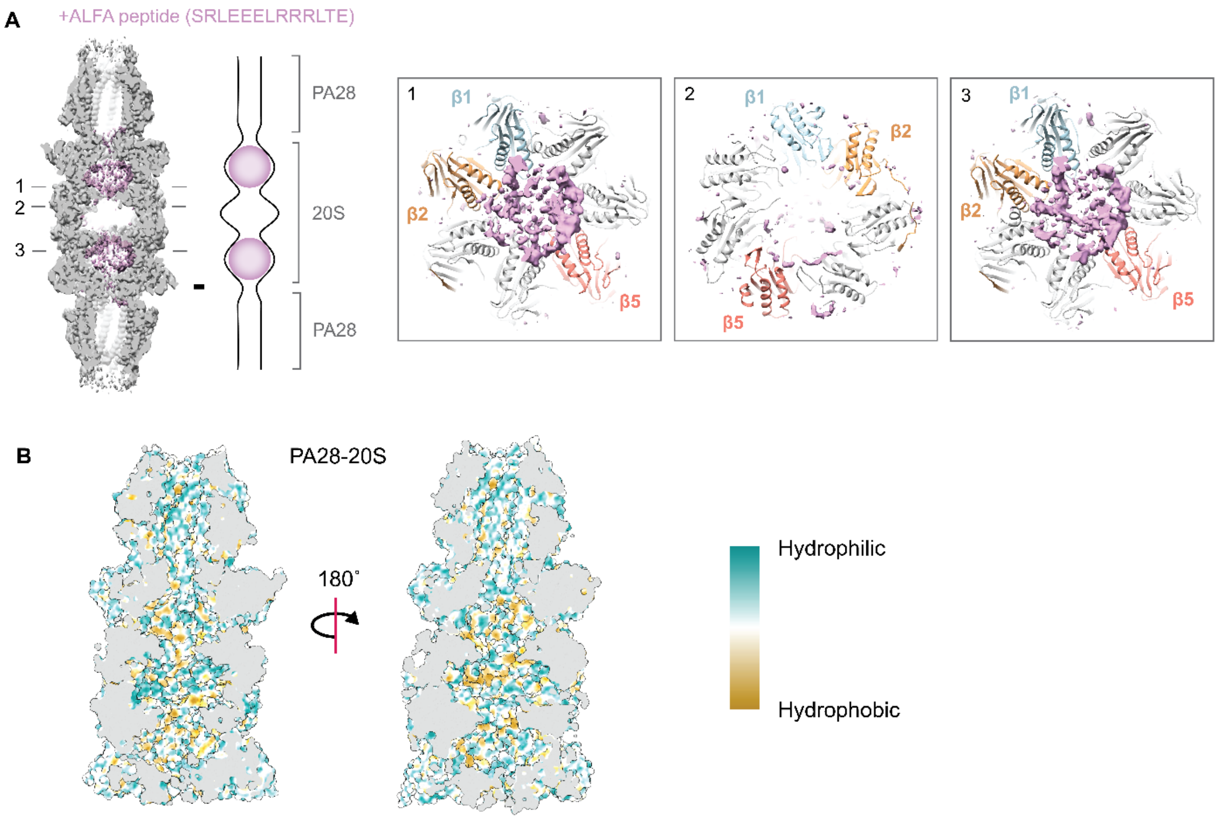

Supplementary Figure 7. (**A**) Cryo-EM map of double capped PA28-20S-PA28 shows ALFA peptide density in both antechambers. (**B**) The walls of the 20S proteasome inner chambers are made up of both hydrophilic and hydrophobic regions.

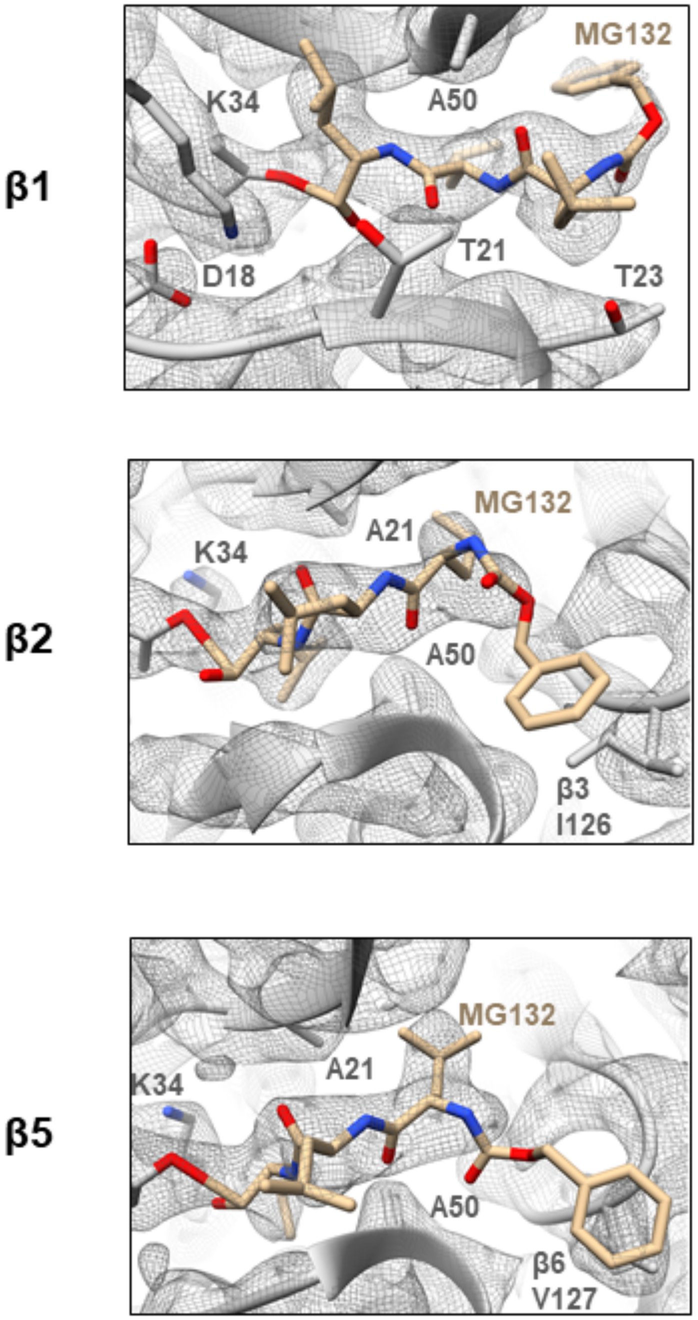

Supplementary Figure 8. Fit of MG132 into the PA28-20S cryo-EM density at the proteolytic sites of β1, β2, and β5.

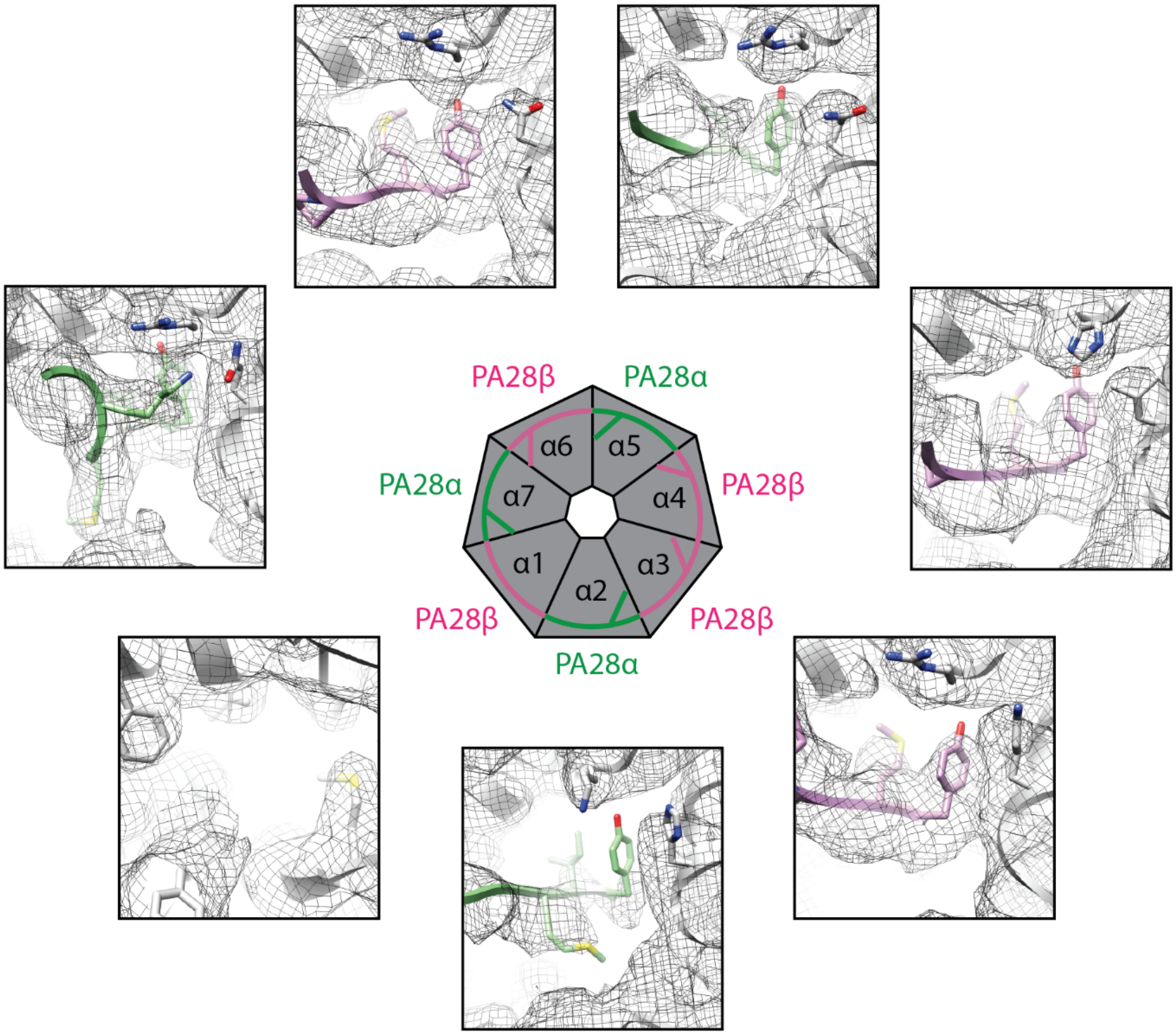

Supplementary Figure 9. PA28 C-terminal tail model and map density. Density is observed for the C-terminal tails of PA28 subunits for 6 out of 7 binding sites.

Supplementary Table 1. Cryo-EM imaging parameters

**PSMB2-StrepII pulldown**	
Microscope	Polara (300 kV), UCSF
Camera (software)	Gatan K2 Summit (SerialEM, 1 shot/hole)
Magnification (at detector)	40,984X
Exposure rate (total)	8 e/pixel/s (43 e/Å^2)
Frames	30
Pixel size	1.22 Å/pixel
Defocus range (um)	1.2 – 3.1
Micrographs (# initial particles)	3761 (1,912,794)
# of 3D classes (# particles)	3 (550,396)
Class 1 (# particles), codes<br>Resolution (0.143/0.5)<br>Local resolution (min-max)<br>3DFSC spread (0.143)<br>Sharpening B-factor	20S (499,629), EMD-24275, PDB 7NAN<br>2.8/3.8 A (mask), 3.5/4.1 A (no mask)<br>2.7 – 29 A<br>2.5 – 2.8A<br>0
Class 2 (# particles), codes<br>Resolution (0.143/0.5)<br>Local resolution (min-max)<br>3DFSC spread (0.143)<br>Sharpening B-factor	PA200-20S (50,767), EMD-24278, PDB 7NAQ<br>3.2/4.4 A (mask), 4.2/7.6 A (no mask)<br>2.8 – 50 A<br>3.0 – 3.6 A<br>0
Class 3 (# particles)	19S-20S (did not refine)
**ALFA-PSME1 pulldown**	
Microscope	Polara (300 kV), UCSF
Camera (software)	Gatan K2 Summit (SerialEM, 1 shot/hole)
Magnification (at detector)	40,984X
Exposure rate (total)	8 e/pixel/s (43 e/Å^2)
Frames	30
Pixel size	1.22 Å/pixel
Defocus range (um)	1.3 – 2.0
Micrographs (# initial particles)	5994 (2,540,092)
# of 3D classes (# particles)	2 (158,883)
Class 1 (# particles), codes<br>Resolution (0.143/0.5)<br>Local resolution (min-max)<br>3DFSC spread (0.143)<br>Sharpening B-factor	PA28-20S (135,937), EMD-24276, PDB 7NAO<br>2.9/3.9 A (mask), 3.8/5.3 A (no mask)<br>2.7 – 43 A<br>2.6 – 3.2 A<br>0
Class 2 (# particles), codes<br>Resolution (0.143/0.5)<br>Local resolution (min-max)<br>3DFSC spread (0.143)<br>Sharpening B-factor	PA28-20S-PA28 (22,946), EMD-24277, PDB 7NAP<br>3.2/4.5 A (mask), 6.1/8.5 A (no mask)<br>2.7 – 52 A<br>3.0 – 3.4 A<br>0
**PSMB2-StrepII pulldown + MG132**	
Microscope	Titan Krios (300 kV), SBP
Camera (software)	Gatan K3 (SerialEM, 4-shots/hole)

Magnification (at detector)	47,170X
Exposure rate (total)	25 e/pixel/s (30 e/Å^2)
Frames	20
Pixel size	1.06 Å/pixel
Defocus range (um)	1.3 – 2.3
Micrographs (# initial particles)	8128 (3,537,033)
# of 3D classes	7 (729,026)
Class 1 (# particles), codes<br>Resolution (0.143/0.5)<br>Local resolution (min-max)<br>3DFSC spread (0.143)<br>Sharpening B-factor	20S (214,790), EMD-27013, PDB 8CVR<br>2.7/3.1 A (masked), 3.3/4.1 A (unmasked)<br>2.5 – 45 A<br>2.5 – 2.8 A<br>0
Class 2 (# particles), codes<br>Resolution (0.143/0.5)<br>Local resolution (min-max)<br>3DFSC spread (0.143)<br>Sharpening B-factor	PA200-20S (70,412), EMD-27015, PDB 8CVS<br>3.1/4.3 A (masked), 4.1/8.0 (unmasked)<br>2.4 – 40 A<br>2.2 – 3.6 A<br>0
Class 3 (# particles), codes<br>Resolution (0.143/0.5)<br>Local resolution (min-max)<br>3DFSC spread (0.143)<br>Sharpening B-factor	PA28-20S (136,392), EMD-27014<br>2.8/3.4 A (masked), 4.1/7.8 A (unmasked)<br>2.6 – 45 A<br>2.3 – 3.0 A<br>0
Class 4 (# particles), codes<br>Resolution (0.143/0.5)<br>Local resolution (min-max)<br>3DFSC spread (0.143)<br>Sharpening B-factor	19S-20S, S_A/E_A (65,390), EMD-27016<br>3.3/4.1 A (masked), 7.1/9.4 A (unmasked)<br>3.1 – 57 A<br>3.0 – 4.0 A<br>0
Class 5 (# particles), codes<br>Resolution (0.143/0.5)<br>Local resolution (min-max)<br>3DFSC spread (0.143)<br>Sharpening B-factor	19S-20S, S_{D1} (45,734), EMD-27017<br>3.4/4.2 A (masked), 7.3/11 A (unmasked)<br>3.1 – 57 A<br>3.2 – 3.9 A<br>0
Class 6 (# particles), codes<br>Resolution (0.143/0.5)<br>Local resolution (min-max)<br>3DFSC spread (0.143)<br>Sharpening B-factor	19S-20S, S_{D2}/E_{D2} (147,696), EMD-27018, PDB 8CVT<br>3.1/3.8 A (masked), 4.7/8.9 A (unmasked)<br>2.8 – 51 A<br>2.2 – 4.0 A<br>0
Class 7 (# particles), codes<br>Resolution (0.143/0.5)<br>Local resolution (min-max)<br>3DFSC spread (0.143)<br>Sharpening B-factor	19S-20S, S_{D3}/E_{C2} (48,612), EMD-27019<br>3.3/4.1 A (masked), 7.2/11 A (unmasked)<br>3.1 – 57 A<br>3.1 – 3.8 A<br>0

Supplementary Table 2. Comparing PA28(4a3b) and PA28(3a4b) models

Model composition	20S-PA28(4a3b)	20S-PA28(3a4b)
PDB code	8CXB	7NAO
Atomic modeling package	Coot	Coot
CCvolume/CCmask	0.85/0.85	0.85/0.85
B-factor (masked/unmasked)	70/75	70/75
RMSD bond lengths, angles	0.007/1.281	0.006/1.090
Molprobity score	1.41	1.04
Clashscore	4.59	2.51
Rotamers outliers (%)	1.71	0
C-beta outliers (%)	0.25	0
Ramachandrans (Favored, outliers) %	98.57/0.09	98.63/0.07
CaBLAM outliers (%)	1.39	1.41
EMRinger score	3.05	3.09
